# Hunger Tradeoffs and Coping Strategies Among Families with Food Insecurity in Massachusetts

**DOI:** 10.3390/nu18132087

**Published:** 2026-06-26

**Authors:** Cara F. Ruggiero, Man Luo, Meghan Perkins, Catherine D. Lynn, Daniel Taitelbaum, Cheryl Schondek, Christopher R. Long, Stephanie Ettinger De Cuba, Elena Byhoff, Lauren Fiechtner

**Affiliations:** 1 Division of General Academic Pediatrics, Department of Pediatrics, Mass General Brigham for Children, 125 Nashua Street, Boston, MA 02114, USAmluo1@mgb.org (M.L.); meperkins@mgb.org (M.P.);; 2 Communication and Public Affairs, the Greater Boston Food Bank, 70 South Bay Ave, Boston, MA 02118, USA; 3 Business and Data Analytics Department, the Greater Boston Food Bank, 70 South Bay Ave, Boston, MA 02118, USA; dtaitelbaum@gbfb.org (D.T.); cschondek@gbfb.org (C.S.); 4 Center for Nutrition and Health Impact, 20603 Elkhorn Drive, Omaha, NE 68022, USA; longcr@gmail.com; 5 Boston University School of Public Health, Boston University Chobanian and Avedisian, 715 Albany Street, Boston, MA 02118, USA; sedc@bu.edu; 6 Department of Medicine, UMass Chan Medical School, 55 Lake Ave N, Worcester, MA 01655, USA; 7 Division of Pediatric Gastroenterology and Nutrition, Mass General Brigham for Children, 55 Fruit Street, Boston, MA 02114, USA

**Keywords:** food insecurity, tradeoffs, coping strategies, food assistance programs

## Abstract

**Background/Objectives**: Many individuals facing food insecurity make tradeoff decisions between spending money on food or other basic needs. In addition, many engage in coping strategies to stretch food budgets or make food last longer. There is limited data on how participation in food assistance programs is associated with use of tradeoffs or coping strategies among households experiencing food insecurity. **Methods**: A secondary analysis of 2021, 2022 and 2023 cross-sectional state-wide survey on food access, including questions on use of tradeoffs and coping mechanisms, and participation in food assistance programs (Special Supplemental Nutrition Program for Women, Infants, and Children, Supplemental Nutrition Assistance Program, school meals or food pantries). Prevalences of tradeoffs and coping strategies by food assistance use were determined. Multivariable logistic regression models determined odds of tradeoffs and coping strategies reported by food-insecure households. **Results**: Overall, 85% of respondents reported making any tradeoffs and 90% reported any coping strategy use in the prior year. Any food assistance program participation was associated with higher prevalences of making any tradeoff compared to not participating in food assistance programs (89% vs. 78%, *p* < 0.001) and a higher prevalence of engaging in any coping strategy (92% vs. 84%, *p* < 0.001). Food assistance participation was associated with increased odds of making any tradeoff (OR 1.82 95% CI 1.39, 2.39) or any coping strategy (1.85, 95% CI 1.22, 2.80). **Conclusions**: Meeting food assistance program income eligibility requirements is likely a marker of poverty that extends beyond food insecurity. Food assistance is necessary but insufficient to meet family needs.

## 1. Introduction

Rates of food insecurity have been steadily climbing in the U.S. and are highest in low-income, racial and ethnic minoritized groups, and single parent households [1]. Food insecurity regularly co-occurs with household economic hardships that require tradeoffs [2]. Tradeoffs are survival mechanisms used when individuals have to make decisions on how to allocate limited resources [3]. Food-insecure households frequently need to make tradeoff decisions between paying for food and other needs. As household expenses rise, individuals and families are increasingly making tradeoffs between food and other necessary expenses like utilities, housing or medical care [4,5]. Tradeoff decision-making while experiencing food insecurity also contributes to poor diet quality and exacerbates stark disparities in chronic diseases [6,7,8]. Understanding the frequency of tradeoffs among households facing food insecurity can be informative regarding the ripple effects of food insecurity on other unmet basic needs.

In addition to tradeoffs, people facing food insecurity employ coping strategies—behavior modifications to manage the consequences of food insecurity and avoid hunger. Coping strategies include skipping meals, sharing meals, or watering down food or drinks to make food last longer. Tradeoffs and coping strategies are common among food-insecure households, as food budgets are often the first to get cut when facing economic adversity [9]. Recent studies found that rural food-insecure households make frequent tradeoffs, with the highest number among those reporting very low food security [10,11]. Understanding how food insecurity is associated with tradeoffs and coping strategies may help to prioritize policies to address poverty and reduce the rising burden of chronic disease [2,12,13].

Demand for food assistance has increased substantially since 2020, when COVID-19 economic disruptions intensified existing financial challenges and introduced new hardships [14,15]. As with other major economic disruptions, those with the lowest income recover the slowest [16]. As food insecurity continues to rise across the U.S., there has been a compensatory increase in the use of charitable food assistance programs, like food banks and federal food assistance enrollment, including the Supplemental Nutrition Assistance Program (SNAP) and the Special Supplemental Nutrition Program for Women, Infants and Children (WIC) [17,18,19]. Despite these trends, there has been little research looking at tradeoffs and coping strategy use across state-wide populations or among those accessing food assistance. The purpose of this study was to (1) describe the prevalence of tradeoffs and coping strategies among food-insecure individuals across Massachusetts and (2) determine the effects of food assistance program participation on engaging in tradeoffs or coping strategies.

## 2. Materials and Methods

### 2.1. Study Population

Since 2020, The Greater Boston Food Bank and Mass General Brigham conduct an annual cross-sectional survey to assess food access and equity in Massachusetts households [20]. The survey is offered in English, Spanish, Portuguese, Simplified Chinese and Haitian Creole with a focus on experiences with food access in the past year. It is revised and adapted every year with input from state government agencies and community and healthcare partners. Participants are recruited to complete the survey online through the Qualtrics Panels Project [20]. Adults aged 18 years or older, living in Massachusetts, and able to complete the online survey are eligible. Participants confirm their eligibility and consent via self-reported questions within Qualtrics. After being presented with a Fact Sheet describing the study, participants are asked the following and must select *yes* to proceed with the survey, “Do you agree to participate in this study and confirm that you are 18 years of age or older?” Survey design includes response quotas for income, gender, race, ethnicity, age, education, and region (Boston metro, Northeast MA, Southeast MA, Central MA, Western MA) to ensure broad representation across the Massachusetts population with applied weighting methods to produce estimates reflective of the state’s overall demographics with intentional oversampling of low-income individuals to enable a focus on food security and food access for low-income households with an annual sample size goal of at least 3000 high-quality responses [20]. The analysis includes all survey respondents reporting experiences of food insecurity in the 2021, 2022, and 2023 survey waves. The Mass General Brigham Institutional Review Board deemed this study to be exempt (Protocol: 2021P003193, approval date: 16 November 2021), and the study was conducted in accordance with all institutional and research guidelines. Respondents are asked to report their zip code to study regional differences within the state of Massachusetts, but no other potentially identifiable information is collected.

### 2.2. Outcomes

*Tradeoffs* were assessed by five questions about economic tradeoffs made during the past year, which were choosing between paying for food and each of the following: (1) medical care, (2) utilities, (3) rent/mortgage, (4) transportation, or (5) education expenses. Response options included: Every month, Some months during the year, 1 or 2 times per year coded as yes, and Never coded as no. Missingness for this outcome was 0.05% of the sample. *Coping strategies* were assessed by questions with yes/no responses on the following questions: In the past 12 months I/my family, (1) received help from family or friends; (2) sold or pawned personal property; (3) bought the cheapest food available; (4) watered down food or drinks (or watered down infant formula among households with children under 5). Data on coping strategies were only collected in 2022 and 2023. Tradeoff and coping strategy questions are based on those asked in the Hunger in America Survey from Feeding America, and variables were analyzed as binary variables for each tradeoff or coping strategy [2]. We created binary count variables for reporting any tradeoff (yes/no) or coping strategy (yes/no) and count variables for number of reported tradeoffs and number of reported coping strategies. As a sensitivity analysis we included tradeoff and coping strategy scales, a validated measure, where the final tradeoff scale is 5–20 based on Likert scale responses (Never = 1, Every month = 4), and the final coping strategy scale is 0–4 based on yes(=1)/no(=0) responses to each question [21].

### 2.3. Exposures

To explore participation in food assistance programs, we constructed two categories of food programs: federal nutrition programs and food pantries.

We assessed federal nutrition program participation using survey items that asked respondents whether they or a household member had participated in the Supplemental Nutrition Assistance Program (SNAP) program, the Special Supplemental Nutrition Program for Women Infants and Children (WIC), or free school meals (which is universal in Massachusetts) in the last 30 days, the last year, more than a year ago, or never. We assessed food pantry participation using survey items that asked respondents if they received support from a food pantry in the last 30 days, the last year, more than a year ago, or never. Respondents were considered to have participated in a food pantry if they reported visiting a pantry in the last 30 days or in the last year.

### 2.4. Covariates

Demographic questions included self-identified race/ethnicity, sexual orientation, gender identity, household size, if any children are in the household, and household income. Race/ethnicity was defined according to US Census Bureau methods. We assessed household income using set categories ($10,000 to $24,999; $25,000 to $49,999; $50,000 to $74,999; $75,000 to $99,999; $100,000 to $149,999; $150,000 to $199,999; and ≥$200,000). We assessed food insecurity status using the USDA 6-item short form household food insecurity module for 2021 survey data [22]. The 2022 and 2023 survey data used the USDA 18-item household food insecurity module [22]. All USDA modules categorized responses as very low, low, marginal, and high food security. Very low food insecurity is the most severe category, indicative of skipping meals or not eating for an entire day because the household cannot afford enough food [22].

### 2.5. Statistical Analysis

We conducted analyses using R version 4.1.0 [23]. Statistical significance was defined as *p* < 0.05. A raking procedure generated sampling weights and trimmed weights >5. Weights were calculated using distributions of gender, age group, race/ethnicity, education, income category, and geographic region for adults in Massachusetts obtained from American Community Survey (ACS) data from the United States Census Bureau website and the tidycensus package [20]. The demographic distribution of our survey data was similar to the ACS data, except that our survey data had an overrepresentation of adults with children. Point estimates, confidence intervals, and hypothesis tests were calculated using the survey R package to account for the sampling weights, which were applied to all analyses to ensure results were population-representative estimates. We first determined prevalences of any tradeoffs and coping strategies for all the respondents experiencing food insecurity to estimate state-wide tradeoff and coping strategies overall, and stratified by accessing any food assistance. We then evaluated prevalences of tradeoffs and coping strategies by type of food assistance accessed (SNAP, WIC, school meals, or food pantries). Only families with children under 5 in the household were included in WIC analyses, and families with school-aged children were included in school meals analyses.

We constructed separate logistic regression models for each univariate association between reporting tradeoffs and the independent variables: demographics, participation in any food assistance program, food insecurity status (low or very low food insecurity), and survey year to assess individual predictors of tradeoffs. We also introduced an interaction term between food assistance use and presence of children in the household to assess whether the relationship between food assistance program participation and making any tradeoffs differed between households with vs. without children, as having children informs eligibility for two of the four food assistance programs included in this study (WIC and school meals). Variables that were statistically significantly associated with the outcome in the univariate analysis and income were then included in multivariable logistic regression models. This process was then repeated, using any coping strategy as the outcome.

For each outcome, we also examined effect modification by food insecurity status by including an interaction term between it and any food assistance use. Since the interaction term was not statistically significant, there was no evidence of effect modification and analyses were not stratified by food insecurity status. We conducted additional sensitivity analyses using tradeoff and coping strategy scales instead of as dichotomous measures, using a linear regression to evaluate the associations between continuous tradeoff and coping strategy scores and food assistance program participation.

## 3. Results

Of the 9123 survey respondents, the final study sample included 2970 respondents who reported food insecurity during the 2021, 2022, or 2023 survey years (Table 1). The majority of respondents identified as non-Hispanic White (64%), 44% were between the ages of 35–54 years old, 53% were female, 42% reported children in the household, and 29% had an annual household income <$25,000 with 51% reporting an income <$50,000. More details on respondents included in the final study sample are in Table 1.

Prevalence of tradeoffs and coping strategies are summarized in Table 2 among all survey respondents experiencing food insecurity, by food assistance program participation overall, and by individual food assistance program participation. Eighty-five percent of households in the study sample reported making any tradeoffs, and 90% reported any coping strategy in the past year. Among tradeoffs, choosing between paying for food or utilities was the most prevalent (71%), and the mean number of tradeoffs among respondents was three. Respondents reporting any food assistance program participation had higher prevalences of making any tradeoff compared to those not participating in food assistance programs (89% vs. 78%, *p* < 0.001). Among coping strategies, buying the cheapest available food was the most prevalent (86%). The mean number of coping strategies among respondents was two. Reporting any food assistance program participation was associated with higher prevalence of engaging in any coping strategy (92% vs. 84%, *p* < 0.001).

Table 3 presents odds ratios (OR) of reporting any tradeoffs and any coping strategy. In univariate analyses, participating in any food assistance program (OR 2.27, 95% CI 1.77–2.91), reporting Hispanic or Latino ethnicity (OR 1.60, 95% CI 1.10–2.31), reporting very low food security (OR 4.44, 95% CI 3.45–5.71), and having children in the household (OR 1.48, 95% CI 1.14–1.91) were all associated with statistically significantly higher odds of reporting any tradeoff. Older age (≥65 years) was associated with lower odds of making a tradeoff (OR 0.61, 95% CI 0.41–0.91). In the multivariable model, food assistance participation (OR 1.82, 95% CI 1.39–2.39), reporting Hispanic or Latino ethnicity (OR 1.55, 95% CI 1.03, 2.34), household income <$25,000 (OR 0.49, 95% CI 0.32, 0.76) and experiencing very low food security (OR 4.45, 95% CI 3.45–5.74) remained statistically significantly associated with tradeoffs. All other variables lost significance.

Similarly, odds of reporting any coping strategy were significantly higher for respondents who participated in any type of food assistance (OR 2.08, 95% CI 1.46, 2.96). Compared to the youngest age group, all three age groups were associated with increased odds of coping strategies in the univariate analysis (35–54 years, OR 1.99 [95% CI 1.37–2.90]; 55–64 years, OR 1.76 [95% CI 1.00–3.08]; ≥65 years, OR 1.97 [95% CI 1.14–3.43]). Non-Hispanic Black and Hispanic/Latino respondents had lower odds of reporting coping strategies compared to White respondents (OR 0.36, [95% CI 0.22], 0.59; OR 0.51 [95% CI 0.33, 0.78]; respectively). In multivariable models, only Non-Hispanic Black and Hispanic/Latino, identifying as female, and very low food security remained significantly associated with coping strategies in multivariable models. In sensitivity analyses using tradeoff and coping scores, food assistance participants reported 2.01 more tradeoffs (95% CI 1.58–2.45) and 0.41 more coping strategies (95% CI 0.26–0.56) compared with non-participants. After adjustment for demographic and household characteristics, food assistance participation remained independently associated with higher tradeoff scores (0.92, 95% CI 0.4–1.42) and greater use of coping strategies (0.23, 95% CI 0.09–0.37) (see Supplemental Table S1 for full details).

## 4. Discussion

Results from the current study of food-insecure households across Massachusetts suggest that the prevalence of both making tradeoffs and engaging in coping strategies is high, with 85% of respondents reporting making any tradeoff and 90% reporting using any coping strategy. Participation in any food assistance program, including federal programs like SNAP, WIC or school meals, or community programs like visiting a food pantry, was associated with increased odds of making a tradeoff between paying for food and another basic need as well as increased odds of engaging in coping strategies. This highlights how when households face food insecurity, it is often a symptom of a much deeper poverty, where participating in any type of food assistance does not eliminate the need for other supports. Instead, meeting the low-income qualifications for federal food assistance programs is likely a marker of significant poverty that extends beyond food insecurity.

Similar to prior work, we found that among households that are food insecure, using tradeoff and coping strategies is common, and individuals accessing food assistance are more likely to engage in tradeoffs and coping strategies, especially by purchasing lowest cost foods [10,11,24,25]. Findings from this analysis extends prior work by looking across a representative sample of low-income individuals in a repeated cross-sectional state-wide survey, rather than among current pantry participants [24] or a subset of rural counties [11]. The current study examines specific types of tradeoffs and coping strategies households with food insecurity engage in. Our findings are in line with previous studies that have demonstrated that households experiencing food insecurity were more likely to have to make more tradeoffs and reported challenges paying for utilities, housing, transportation and medical bills compared to food-secure households [10,26]. Findings are also similar to a study by Long et al. that found that survey respondents who reported the more frequent putting off in buying medicine to buy food were more frequent utilizers of food pantries [25].

Massachusetts is a state with generous social support systems and a coordinated charity food system, with centralized food banks and local distribution centers across the state, along with a recent state-wide push to increase SNAP enrollment by linking it to Medicaid insurance enrollment [27]. Despite these food resources, high rates of food insecurity persist [20], suggesting these support systems are necessary but not sufficient to eliminate food insecurity. Findings add nuance to the complex decision-making happening among households with low incomes and the ways in which the household budget is a single entity, not siloed between needs. If one cost increases, there are ripples to other needs. In turn, this highlights how food insecurity exists within a broader context of relationships between basic needs, family circumstances, and prevailing regional costs.

Our study found that food-insecure households participating in WIC, SNAP, or school meals or utilizing food pantries within the past year were more likely to engage in tradeoffs and use coping strategies in the same time frame. These findings are important, as the future of such programs are at risk. Recent legislation, including House Resolution 1, also known as the One Big Beautiful Bill Act, includes funding cuts and new restrictions to federal nutrition programs like SNAP, WIC, and school meals, potentially weakening the safety net for low-income families amid ongoing food insecurity. The relationship between food insecurity and federal nutrition support systems is nuanced and often misinterpreted. Simply put, those most in need of help are motivated to seek help [28], but that help is not enough to solve the problem [29]. Due to these factors, participants in federal nutrition programs may continue to experience food insecurity. The need for ongoing tradeoffs and coping strategies among those who participate in food assistance programs likely suggests that these programs need to be strengthened. During the COVID-19 pandemic, SNAP-enrolled families received increased SNAP benefits, with data suggesting that these enhanced SNAP benefits improved mental health and food insecurity among recipients [30]. The return of benefit structures to pre-pandemic levels highlight how current levels are insufficient to meet the basic financial needs of participating households [31]. Additional complementary approaches may also be needed to support households experiencing food insecurity. One such example is the expanded Child Tax Credit (CTC) during the COVID-19 pandemic that cut child poverty in half [32]. As an income transfer program, families had the freedom to determine how to direct the expanded CTC dollars in their budget, providing a flexibility that was highly valued by recipients. Families who received the expanded CTC reported spending the money on a variety of needs including child care, housing, and food [33]. The expanded CTC demonstrated significant reductions in multiple economic hardships, including household food insecurity, and improvements in children’s diet quality [34,35]. The sunsetting of the expanded CTC in 2022 resulted in a return to pre-pandemic levels of childhood poverty [36] and food insufficiency [37] with recent precarity of federal programs exacerbating food insecurity [36,38].

### Strengths and Limitations

Strengths of this analysis include a large, diverse, state-wide representative sample of adults in Massachusetts as well as novel data characterizing households making tradeoffs and coping strategies. Our findings add to the growing literature on contributors to and consequences of food insecurity. There are limitations to this study worth mentioning. The survey was offered online and was not available to households without access to the internet or a cell phone. By pooling cross-sectional surveys we cannot determine causation or directionality of our findings. That is, those most in need seek out assistance and thus may also be those most likely to struggle with tradeoffs or engage in coping strategies. Lastly, misclassification is a possibility in our exposures and outcomes due to consolidation of categories to make results interpretable. However, we evaluated prevalences prior to consolidation to ensure that condensing the data was not obscuring important distinctions, and ran sensitivity analyses as described.

## 5. Conclusions

Our results highlight the multiple tradeoffs and coping strategies food-insecure households make, especially as many households are feeling the financial effects from inflationary pressures and higher costs of food. Food assistance programs and nutrition assistance policies aiming to improve food security must (1) have benefit structures that adequately match the cost of living and (2) preserve and advance program structures that facilitate enrollment and retention to prevent the need for households to make tradeoffs between basic needs. Increases to SNAP during the pandemic were beneficial, but were combined with other financial supports to reduce poverty. Post-pandemic returns to high rates of childhood poverty with ongoing threats to federal nutrition programs like SNAP and funding cuts to the USDA will impact access to food across the country. A more holistic approach for public policies and programs would address the complicated economic hardships faced by food-insecure households to improve food insecurity and poverty. A simultaneous focus on upstream social needs such as household financial resources and economic mobility has the potential to improve household food security.

## Figures and Tables

**Table 1:** Socio-demographic characteristics of weighted food-insecure households.

	Overall ^1^ (N = 2970)
Race/Ethnicity, n (%)	
Non-Hispanic White	1914 (64.4%)
Non-Hispanic Black	302 (10.2%)
Hispanic or Latino	532 (17.9%)
Non-Hispanic Other	222 (7.5%)
Age, n (%)	
18–34	931 (31.4%)
35–54	1316 (44.3%)
55–64	411 (13.8%)
65 or older	312 (10.5%)
Gender Identity, n (%)	
Female	1588 (53.5%)
Male	1309 (44.1%)
Non-binary/Transgender/Other	73 (2.5%)
Sexual Orientation, n (%)	
Non-LGBTQ+	2409 (81.1%)
LGBTQ+	561 (18.9%)
Households with Children, n (%)	
No	1728 (58.2%)
Yes	1242 (41.8%)
Average number of children ^3^, mean (SD)	2.0 (1.3)
Annual Household Income, n (%)	
<$25,000	845 (28.5%)
$25,000–$49,999	663 (22.3%)
$50,000–$74,999	490 (16.5%)
$75,000–$99,999	303 (10.2%)
≥$100,000	669 (22.5%)
SNAP Participant, n (%)	1318 (44%)
WIC Participant ^2^, n (%)	158 (30%)
School Meals Participant ^3^, n (%)	849 (68%)
Food Pantry Participant, n (%)	1218 (41%)

^1^ Weighted frequencies and percentages are reported in this table. ^2^ Among N = 527 households with children under 5 years. ^3^ Among N = 1242 households with children under 18 years.

**Table 2:** Prevalence of tradeoffs and coping strategies among food-insecure households by food assistance program.

	Overall ^1^	No Food Assistance	Any Food Assistance	*p*-Value ^2^	SNAP	WIC ^3^	School Meals ^4^	Food Pantry
**Tradeoffs**	N = 2970	N = 996	N = 1974		N = 1318	N = 158	N = 849	N = 1218
Any tradeoffs ^7^	2524 (85.4%)	**778 (78.2%)**	**1746 (89.1%)**	<0.001	1167 (89.1%)	148 (93.5%)	773 (92.2%)	1095 (90.9%)
Medicine or medical care tradeoffs	1756 (59.3%)	**490 (49.2%)**	**1266 (64.4%)**	<0.001	817 (62.2%)	125 (78.9%)	595 (70.6%)	855 (70.6%)
Utilities tradeoffs	2114 (71.4%)	**614 (61.6%)**	**1500 (76.4%)**	<0.001	996 (75.8%)	138 (86.9%)	693 (82.5%)	967 (80.0%)
Rent or mortgage tradeoffs	1903 (64.2%)	**520 (52.2%)**	**1383 (70.3%)**	<0.001	934 (71.0%)	133 (83.9%)	629 (74.6%)	903 (74.6%)
Transportation tradeoffs	2030 (68.5%)	**568 (57.1%)**	**1462 (74.3%)**	<0.001	984 (74.7%)	138 (87.3%)	667 (79.0%)	945 (78.0%)
School loans, tuition, education expenses tradeoffs	1294 (43.7%)	**349 (35.1%)**	**944 (48.0%)**	<0.001	598 (45.5%)	124 (78.2%)	516 (61.2%)	636 (52.6%)
# of tradeoffs, mean (SD) ^7^	3.07 (1.82)	**2.55 (1.87)**	**3.33 (1.73)**	<0.001	3.29 (1.73)	4.15 (1.52)	3.67 (1.66)	3.55 (1.67)
Food Insecurity Level								
Very Low	1770 (60.4%)	**480 (48.8%)**	**1290 (66.3%)**	<0.001	936 (71.2%)	118 (74.4%)	534 (64.9%)	842 (69.9%)
Low	1158 (39.6%)	**503 (51.2%)**	**655 (33.7%)**		378 (28.8%)	41 (25.6%)	289 (35.1%)	364 (30.1%)
Number of Tradeoffs ^7^								
0 tradeoff	431 (14.6%)	**217 (21.8%)**	**214 (10.9%)**	<0.001	143 (10.9%)	10 (6.5%)	65 (7.8%)	110 (9.1%)
1 tradeoff	311 (10.5%)	**138 (13.8%)**	**173 (8.8%)**		119 (9.1%)	4 (2.8%)	64 (7.6%)	84 (7.0%)
2 tradeoffs	326 (11.0%)	**125 (12.6%)**	**201 (10.2%)**		142 (10.9%)	11 (6.7%)	67 (8.0%)	102 (8.4%)
3 tradeoffs	390 (13.2%)	**128 (12.9%)**	**262 (13.4%)**		189 (14.4%)	10 (6.0%)	92 (11.0%)	155 (12.9%)
4 tradeoffs	552 (18.7%)	**168 (16.9%)**	**384 (19.6%)**		246 (18.8%)	14 (8.9%)	143 (17.1%)	244 (20.2%)
5 tradeoffs	944 (32%)	**218 (22.0%)**	**726 (37.0%)**		470 (35.9%)	109 (69.0%)	406 (48.5%)	511 (42.4%)
**Hunger Coping Strategies** ^5^	N = 2029	N = 517	N = 1512		N = 1145	N = 141	N = 596	N = 990
Any coping strategy	1824 (89.9%)	**436 (84.3%)**	**1388 (91.8%)**	<0.001	1064 (93.0%)	133 (94.1%)	543 (91.1%)	907 (91.6%)
Received help from family or friends	1089 (60.4%)	**210 (48.1%)**	**879 (64.4%)**	<0.001	704 (66.7%)	95 (70.8%)	351 (64.7%)	585 (66.0%)
Sold or pawned personal property	792 (43.8%)	**163 (36.0%)**	**629 (46.3%)**	<0.001	509 (48.3%)	48 (41.6%)	245 (46.8%)	437 (49.9%)
Bought the cheapest food available	1558 (85.2%)	379 (84.4%)	1179 (85.5%)	0.6	927 (87.2%)	93 (79.6%)	425 (82.3%)	756 (83.9%)
Watered down food or drinks	689 (37.8%)	**128 (28.2%)**	**561 (41.0%)**	<0.001	444 (42.0%)	69 (54.7%)	226 (42.9%)	410 (46.2%)
Watered down infant formula ^6^	44 (28.3%)	5 (18.8%)	39 (30.3%)	0.3	31 (32.1%)	21 (44.3%)	38 (36.2%)	31 (38.1%)
# of coping strategies, mean (SD) ^8^	2.29 (1.09)	**1.97 (1.09)**	**2.39 (1.07)**	<0.001	2.47 (1.07)	2.58 (1.08)	2.41 (1.10)	2.49 (1.09)

^1^ Weighted frequencies and percentages are reported in this table. ^2^ *p* values denote the comparison between no vs. any food assistance. Chi-square tests with Rao & Scott adjustment were performed to compare categorical outcomes, and Design-based Kruskal–Wallis tests were performed for continuous outcomes. ^3^ WIC participation is restricted to households with children under 5 years old (N = 527). ^4^ School Meals participation is restricted to households with children under 18 years old (N = 1242). ^5^ Collected in 2022, 2023 survey waves only. ^6^ Collected in 2023 survey wave only. ^7^ Any tradeoffs and number of tradeoffs are restricted to households with complete answers to all tradeoffs questions (N = 2954, 0.05% missingness). ^8^ Number of coping strategies are restricted to households with complete answers to all coping strategy questions (N = 1563, 23% missingness).

**Table 3:** Odds of making any tradeoff or use of any coping strategy among food-insecure households.

	Tradeoffs Among Food-Insecure Households ^1^		Use of Coping Strategies Among Food-Insecure Households ^3^	
	Univariate	Multivariable ^2^	Univariate	Multivariable ^2^
	OR (95% CI)			
Any Food Assistance (Federal Nutrition or Food Pantry Participation)	**2.27 (1.77, 2.91)**	**1.82** **(1.** **39** **, 2.** **39** **)**	**2.08 (1.46, 2.96)**	**1.85** **(1.** **22** **, 2.** **80** **)**
Age, n (%)				
18–34	Reference	Reference	Reference	Reference
35–54	1.13 (0.84, 1.53)	1.01 (0.73, 1.40)	**1.99 (1.37, 2.90)**	1.49 (0.995, 2.24)
55–64	0.89 (0.63, 1.25)	0.84 (0.57, 1.24)	**1.76 (1.00, 3.08)**	1.09 (0.61, 1.96)
65 or older	**0.61 (0.41, 0.91)**	0.71 (0.45, 1.11)	**1.97 (1.14, 3.43)**	1.50 (0.82, 2.77)
Race/Ethnicity				
Non-Hispanic White	Reference	Reference	Reference	Reference
Non-Hispanic Black	0.94 (0.61, 1.45)	0.88 (0.56, 1.40)	**0.36 (0.22, 0.59)**	**0.33 (0.20, 0.** **57** **)**
Hispanic or Latino	**1.60 (1.10, 2.31)**	**1.55 (1.03, 2.34)**	**0.51 (0.33, 0.78)**	**0.51** **(0.** **32** **, 0.** **82** **)**
Non-Hispanic Other	0.80 (0.48, 1.34)	0.78 (0.47, 1.31)	0.59 (0.34, 1.04)	0.64 (0.36, 1.14)
Household Income				
<$25,000	0.80 (0.52, 1.22)	**0.49 (0.32, 0.76)**	1.00 (0.57, 1.77)	0.71 (0.39, 1.31)
$25,000–$49,999	0.93 (0.59, 1.45)	0.76 (0.49, 1.19)	1.44 (0.81, 2.56)	1.30 (0.71, 2.41)
$50,000–$74,999	0.98 (0.62, 1.55)	0.92 (0.58, 1.46)	1.14 (0.63, 2.08)	1.07 (0.57, 2.01)
$75,000–$99,999	0.69 (0.42, 1.16)	0.68 (0.40, 1.16)	1.04 (0.53, 2.06)	1.05 (0.51, 2.14)
≥$100,000	Reference	Reference	Reference	Reference
Gender Identity				
Male	Reference	-	Reference	Reference
Female	0.97 (0.75, 1.25)	-	**1.55 (1.11, 2.16)**	**1.54** **(1.** **08** **, 2.** **20** **)**
Non-binary/Transgender/Other	0.80 (0.30, 2.12)	-	0.63 (0.25, 1.57)	0.95 (0.30, 3.08)
Sexual Orientation				
Non-LGBTQ+	Reference	-	Reference	-
LGBTQ+	0.87 (0.63, 1.20)	-	1.15 (0.77, 1.71)	-
Household Size	1.07 (0.99, 1.15)	-	**0.92 (0.85, 0.998)**	0.94 (0.87, 1.02)
Survey Year				
2021	Reference	-	-	-
2022	0.80 (0.58, 1.10)	-	Reference	-
2023	0.98 (0.71, 1.35)	-	1.35 (0.96, 1.89)	-
Very Low Food Security	**4.44 (3.45, 5.71)**	**4.45** **(3.** **45** **, 5.** **74** **)**	**4.65 (3.28, 6.58)**	**5.09 (3.54, 7.30)**
Children in Household	**1.48 (1.14, 1.91)**	1.04 (0.77, 1.42)	0.95 (0.67, 1.33)	-
Food Assistance * Children	1.64 (0.93, 2.91)	-	1.43 (0.65, 3.13)	-

^1^ Multivariable models include any significant factor in univariate model. ^2^ This analysis only includes households with complete answers to all tradeoffs questions. Around 0.5% (N = 16) of respondents were excluded due to missingness in tradeoffs questions. ^3^ Collected in 2022, 2023 survey waves only. * It is an interaction term between the food assistance variable and children in the house variable.

## Data Availability

Data will be shared upon request. Proposals can be directed to meperkins@mgh.harvard.edu. To gain access, data requestors need (1) to present a proposal approved by the PI to ensure it falls under the scope of the IRB and the topic is not currently in progress by another investigator, (2) to obtain confirmation of IRB approval from their home institution for the data sharing and analysis, and (3) to complete a data use agreement between Mass General Brigham and their home institution.

## Supplementary Materials

The following supporting information can be downloaded at https://www.mdpi.com/article/10.3390/nu18132087/s1. Table S1: Univariate and Multivariable Linear models evaluating the association between tradeoff and coping strategy scores with any food assistance use.

## Abbreviations

The following abbreviations are used in this manuscript:

SNAP	Supplemental Nutrition Assistance Program
WIC	Special Supplemental Nutrition Program for Women Infants and Children
USDA	United Stated Department of Agriculture
ACS	American Community Survey
CTC	Child Tax Credit
