# Supplementary material for: Hunger Tradeoffs and Coping Strategies Among Families with Food Insecurity in Massachusetts
Source: Nutrients. 2026 Jun 26;18(13):2087. doi: 10.3390/nu18132087 (PMC13363607; doi:10.3390/nu18132087)
Supplement: Supplementary file 1 [file nutrients-18-02087-s001.zip › nutrients-4362618-supplementary.pdf]

Supplementary Table S1: Univariate and Multivariable Linear models evaluating the association between tradeoff and coping strategy scores with any food assistance use

	**Tradeoffs among food insecure households[2]**		**Use of coping strategies among food insecure households[3]**	
	Univariate	Multivariable[1]	Univariate	Multivariable[1]
	Mean difference (95%CI)			
Any food assistance (Federal Nutrition or Food Pantry Participation)	**2.01 (1.58, 2.45)**	**0.92 (0.41, 1.42)**	**0.41 (0.26, 0.56)**	**0.23 (0.09, 0.37)**
Age				
18-34	[Reference]	[Reference]	[Reference]	[Reference]
35-54	0.08 (-0.40, 0.55)	-0.31 (-0.76, 0.14)	-0.002 (-0.14, 0.14)	-0.01 (-0.14, 0.11)
55-64	**-1.19 (-1.78, -0.60)**	**-1.19 (-1.76, -0.62)**	-0.17 (-0.34, 0.001)	-0.16 (-0.32, 0.002)
65 or older	**-2.60 (-3.30, -1.90)**	**-2.22 (-2.88, -1.55)**	**-0.45 (-0.67, -0.23)**	**-0.29 (-0.50, -0.08)**
Race/Ethnicity				
Non-Hispanic White	[Reference]	[Reference]	[Reference]	[Reference]
Non-Hispanic Black	0.62 (-0.12, 1.35)	-	-0.16 (-0.38, 0.06)	-
Hispanic or Latino	1.47 (0.93, 2.01)	-	0.12 (-0.04, 0.27)	**-**
Non-Hispanic Other	0.55 (-0.34, 1.44)	-	0.0004 (-0.28, 0.28)	-
Household Income				
< $25,000	**-0.77 (-1.48, -0.07)**	**-1.20 (-1.85, -0.55)**	0.09 (-0.14, 0.32)	0.004 (-0.020, 0.21)

$25,000-$49,999	-0.68 (-1.40, 0.04)	**-0.67 (-1.33, -0.02)**	0.10 (-0.13, 0.33)	0.15 (-0.05, 0.35)
$50,000-$74,999	-0.72 (-1.46, 0.01)	-0.59 (-1.24, 0.07)	-0.02 (-0.25, 0.21)	0.05 (-0.16, 0.26)
$75,000-$99,999	**-1.44 (-2.27, -0.61)**	**-1.30 (-2.06, -0.54)**	-0.06 (-0.33, 0.21)	0.06 (-0.18, 0.30)
≥ $100,000	[Reference]	[Reference]	[Reference]	[Reference]
Gender Identity				
Male	[Reference]	[Reference]	[Reference]	[Reference]
Female	**-0.62 (-1.05, -0.20)**	**-0.47 (-0.86, -0.07)**	-0.03 (-0.15, 0.10)	-
Non-binary/ Transgender/ Other	-0.04 (-1.51, 1.44)	-0.59 (-1.99, 0.81)	-0.49 (-0.07, 1.05)	**-**
Sexual Orientation				
Non-LGBTQ+	[Reference]	-	[Reference]	-
LGBTQ+	0.35 (-0.19, 0.89)	-	0.33 (0.18, 0.48)	-
Household size	**0.21 (0.12, 0.31)**	0.01 (-0.09, 0.12)	**0.04 (0.01, 0.07)**	0.02 (-0.02, 0.06)
Survey Year				
2021	[Reference]	-	-	-
2022	-0.52 (-1.06, 0.03)	-	[Reference]	[Reference]
2023	-0.47 (-1.00, 0.06)	-	**0.23 (0.11, 0.35)**	**0.21 (0.10, 0.33)**
Very low food insecurity	**2.72 (2.31, 3.12)**	**2.55 (2.16, 2.94)**	**0.76 (0.64, 0.87)**	**0.70 (0.58, 0.82)**
Children in household	**1.27 (0.85, 1.69)**	-0.82 (-1.67, 0.02)	0.10 (-0.02, 0.23)	-

Food assistance*Children	**2.72 (2.31, 3.12)**	**1.54 (0.60, 2.48)**	0.22 (-0.15, 0.59)	-

[1]Multivariable models include any significant factor in univariate models.

[2]This analysis only includes households with complete answers to all tradeoffs questions. Around 0.5% (N=16) of respondents were excluded due to missingness in tradeoffs questions.

[3]Coping strategies data were collected in 2022, 2023 survey waves only. This analysis only includes households who answered "Yes" or "No" to all coping strategies questions. Around 23% (N=466) of respondents were excluded due to missingness in coping strategies questions.
